# Performance of Direct Identification and EUCAST Rapid Antimicrobial Susceptibility Testing in Bloodstream Infections Using Sepsityper^®^ and Disk Diffusion Methods

**DOI:** 10.3390/diagnostics16203302

**Published:** 2026-10-11

**Authors:** Canberk Çınar, Yeliz Tanrıverdi Çaycı, İlknur Bıyık, Ayşegül Çolak, Demet Gür Vural, Kemal Bilgin, Asuman Birinci

**Affiliations:** Department of Medical Microbiology, Faculty of Medicine, Ondokuz Mayıs University, 55139 Samsun, Turkey; yeliztanriverdi@gmail.com (Y.T.Ç.); ilknurbiyik1@hotmail.com (İ.B.); aysegulcolaak@gmail.com (A.Ç.); demet-gur@yandex.com (D.G.V.); kemal.bilgin@omu.edu.tr (K.B.); asumanbirinci@yahoo.com (A.B.)

**Keywords:** Sepsityper, MALDI-TOF MS, rapid antimicrobial susceptibility testing, sepsis, categorical agreement

## Abstract

**Background/Objectives:** Rapid pathogen identification and targeted antimicrobial susceptibility testing (AST) in bloodstream infections are critical to reducing sepsis-related mortality. This study aimed to evaluate the performance of direct MALDI-TOF MS identification using the rapid Sepsityper^®^ kit and to determine the diagnostic accuracy and reliability of the EUCAST Rapid Antimicrobial Susceptibility Testing (RAST) protocol at various incubation periods (4, 6, 8, and 16–20 h) from positive blood culture bottles. **Methods:** A total of 71 clinical isolates recovered from positive blood cultures in the Medical Microbiology Laboratory of Ondokuz Mayıs University Faculty of Medicine were included. Samples prepared directly using the rapid Sepsityper^®^ kit were identified via MALDI-TOF MS, while the EUCAST RAST protocol was simultaneously performed on Mueller–Hinton agar using disk panels guided by Gram stain results. Results were analyzed for Categorical Agreement (CA), major errors, and very major errors using routine automated AST data as the reference standard. **Results:** The rapid Sepsityper^®^ kit successfully identified 51 of 71 isolates (71.8%) at the species level within 30–60 min. The overall categorical agreement of EUCAST RAST with the automated system increased from 67.5% at 4 h to 76.6% at 6 h, 82.7% at 8 h, and 87.0% at 16–20 h. With prolonged incubation, the major error rate dropped significantly from 51.3% to 3.8%. However, the very major error rate remained at 8.1% in final readings, with these errors predominantly clustering in carbapenems and fluoroquinolones among *Klebsiella pneumoniae* strains. **Conclusions:** The rapid Sepsityper^®^ kit provides species-level identification in under an hour in approximately three-quarters of cases, facilitating the early de-escalation of empirical therapy. Although EUCAST RAST categorical agreement improves progressively with time, conventional methods are still recommended for confirmation due to the risk of very major errors, particularly in detecting carbapenem resistance.

## 1. Introduction

Bloodstream infections (BSIs) and sepsis are time-sensitive, emergency clinical conditions characterized by high morbidity and mortality rates [1]. Given the time-critical nature of these conditions, prompt initiation of effective antimicrobial therapy is essential for improving clinical outcomes. In clinical practice, empirical antimicrobial therapy is initiated when infection is suspected, often before microbiological results become available. Therefore, selecting an appropriate empirical antibiotic regimen is crucial, as inappropriate initial antimicrobial therapy may lead to treatment failure and is associated with an increased risk of mortality [2]. Conversely, the prolonged and uncontrolled use of non-targeted broad-spectrum antimicrobial agents leads to the selection of resistant strains and the spread of nosocomial infections [3]. In the conventional microbiological workflow, recovering the pathogen from a positive blood culture, identifying it at the species level, and obtaining standard antimicrobial susceptibility testing (AST) results typically require a timeframe of 24 to 72 h [4]. This delay leads to inadequate patient treatment, particularly when the causative agents are multidrug-resistant (MDR) Gram-negative bacilli or methicillin-resistant *Staphylococcus aureus* (MRSA) strains [5]. In recent years, advancements in proteomic and molecular technologies have provided opportunities for rapid diagnosis directly from blood culture broths [6]. The rapid Sepsityper^®^ kit (Bruker Daltonics) removes human blood cells (erythrocytes/leukocytes) and media components from positive blood culture broth via lysis and centrifugation steps, extracts bacterial proteins, and enables species-level identification within 30–60 min using Matrix-Assisted Laser Desorption/Ionization Time-of-Flight Mass Spectrometry (MALDI-TOF MS) [7]. Simultaneously, the European Committee on Antimicrobial Susceptibility Testing (EUCAST) has established standards for rapid antimicrobial susceptibility testing (RAST) performed directly from positive blood cultures [8]. In this method, positive blood culture broth is directly inoculated onto Mueller–Hinton agar, and categorical evaluation is performed at the end of 4, 6, 8, and 16–20 h incubation periods using species-specific breakpoints [9]. While rapid identification systems significantly shorten the initial diagnostic timeline, coupling them directly with real-time susceptibility testing creates a more comprehensive workflow to address both pathogen profiling and resistance detection simultaneously. Assessing these integrated protocols across multiple different incubation intervals is crucial for defining the optimal balance between rapid clinical turnaround and diagnostic accuracy. The aim of this study is to evaluate the success of species-level identification using the rapid Sepsityper^®^ kit from positive blood culture samples in the Medical Microbiology Laboratory of Ondokuz Mayıs University Faculty of Medicine, and to determine the categorical agreement (CA), major error, and very major error rates by comparing the performance of the simultaneously applied EUCAST RAST protocol at 4, 6, 8, and 16–20 h with routine automated system results.

## 2. Materials and Methods

This study was conducted with 71 clinical isolates obtained from positive blood culture samples sent from the clinics of Ondokuz Mayıs University Faculty of Medicine Hospital to the Medical Microbiology Laboratory, which flagged positive in the BD BACTEC™ FX (Becton Dickinson, Sparks, MD, USA). automated blood culture system. The study evaluated consecutive, non-duplicate clinical blood culture bottles processed using BD BACTEC PLUS Aerobic/F and Peds Plus/F vials (Becton Dickinson, Sparks, MD, USA) Routine identification, automated antimicrobial susceptibility data, and the rapid Sepsityper and EUCAST RAST protocols were evaluated as bottles flagged positive. To exclude duplicate samples from the analysis, only the first positive blood culture from the same patient was included in the study. The strains included in the study are presented in Table 1.

Gram staining was performed first on the blood culture bottles that gave a positive signal in the BD BACTEC™ FX system. Subsequently, 1 mL of positive blood culture broth was taken and subjected to the rapid Sepsityper^®^ kit (Bruker Daltonics, Bremen, Germany) protocol. Human cells were eliminated using lysis buffer, and following washing with washing buffer, bacterial extraction was performed by adding formic acid and acetonitrile onto the obtained pellet. The extract was spotted onto a MALDI-TOF MS target plate, mixed with alpha-cyano-4-hydroxycinnamic acid (HCCA) matrix, and analyzed using a Microflex LT (Bruker Daltonics) device. Target spectra were processed utilizing the MALDI Biotyper Compass software (version 4.1, Bruker Daltonics, Bremen, Germany) and the MBT BDAL reference library (version 11, Bruker Daltonics, Bremen, Germany). In accordance with the manufacturer’s recommendations, MALDI-TOF MS score values were categorized as high-reliability species-level identification (≥2.0), reliable genus-level identification (1.7–1.99), and non-reliable or failed identification (<1.7). MALDI-TOF MS score values were categorized in accordance with the manufacturer’s recommendations as high-reliability species-level identification, genus-level identification, and non-reliable identification. The positive blood culture broth was directly inoculated onto MuellerHinton agar (MHA) plates as a standard inoculum based on the Gram stain result. Specific antibiotic disk panels recommended in the EUCAST RAST guidelines for Gram-negative and Gram-positive microorganisms were placed onto the plates. The plates were incubated aerobically at 35 ± 1 °C, and inhibition zone diameters were measured in millimeters at 4, 6, 8, and 16–20 h. The obtained zone diameters were classified according to species- and reading time-specific breakpoints from the EUCAST RAST guidelines: Susceptible (S), Susceptible, increased exposure/Area of Technical Uncertainty (ATU/I), and Resistant (R). In accordance with EUCAST instructions, 4 h readings were not performed for *P. aeruginosa* isolates. Routine automated AST (VITEK 2, bioMérieux, Marcy-l’Étoile, France/BD Phoenix, Becton Dickinson, Sparks, MD, USA) results performed simultaneously for each isolate were accepted as the reference standard. Six antibiotic-time measurements (*S. aureus*, 4 h reading) whose zone diameters were marked as uncertain and carried a risk of erroneous measurement were excluded from the analysis.

Test performances were evaluated according to the following formulas:

“CA (%) = (number of concordant results/total number of evaluable tests) × 100 (same format for VME and ME)” × 100

“Very Major Error (%)” = “Number of Reference Resistant/RAST Susceptible”/“Total Number of Reference Resistant” × 100

“Major Error (%)” = “Number of Reference Susceptible/RAST Resistant”/“Total Number of Reference Susceptible” × 100

Each RAST result read at 4, 6, 8 and 16–20 h was compared with the reference (routine automated AST) result for the same isolate–antibiotic pair. A test was considered evaluable if a reference category (S, I or R) and a valid RAST zone reading at that time point were both available, after exclusion of the measurements mentioned above. Discordant tests comprised very major errors (VME; reference R, RAST S), major errors (ME; reference S, RAST R) and other discordant results (RAST results in the ATU, and discordances involving an intermediate reference category). An ATU result was classified as discordant when the reference category was S or R, and as concordant when the reference category was I; ATU results were retained in the analysis and not excluded. Concordant tests were calculated as evaluable tests minus discordant tests. VME and ME rates were calculated using, as denominators, all evaluable tests with a reference category of R and S, respectively, including those read as ATU by RAST; because ATU results are already contained within these denominators, they are not additive to them. During the preparation and drafting of this manuscript, AI-based assistance (Google Gemini) was utilized to refine the language, structure the scientific text, and assist with editorial formatting.

## 3. Results

Of the 71 positive blood culture samples included in the study, accurate species-level identification was achieved in 51 (71.8%) within 30–60 min using the rapid Sepsityper^®^ kit and MALDI-TOF MS combination. In the 20 isolates (28.2%) where identification failed, species information was provided by conventional culture and automated systems, and RAST panels were successfully performed guided by Gram stain results. The species-level identification performance of the rapid Sepsityper^®^ kit is presented in Table 2.

A total of 2094 evaluable antibiotic–time-point comparisons were performed across four different time intervals. Evaluable tests were defined as tests with a reference category (S, I or R) and a valid RAST reading.As the incubation time increased, the overall categorical agreement increased steadily and reached 87.0% in the final reading. The overall categorical agreement and test numbers according to time are presented in Table 3.

In the species-based analyses, *Acinetobacter* spp. strains demonstrated high agreement above 90% at all time points, whereas the agreement for *S. aureus* strains, which was 23.1% at 4 h, increased to 93.3% at 16–20 h, reaching the clinical reliability level. Categorical agreement rates according to species are presented in Table 4.

The major error (false resistance) rate, which was as high as 51.3% at the 4 h reading, showed a dramatic decrease as incubation progressed, dropping to 3.8% at the final reading. In contrast, the very major error (false susceptibility) rate peaked at 11.1% at 8 h and was 8.1% at the 16–20 h reading. Distribution of error types according to reading times and the composition of the denominators is presented in Table 5.

The detailed distribution of the 22 very major errors detected in the 16–20 h final reading by species and antibiotic groups is presented in Table 6. It was observed that 73% (16/22) of the very major errors clustered in *K. pneumoniae* isolates, particularly involving carbapenems and fluoroquinolones.

## 4. Discussion

Reducing the time to transition from empirical to targeted therapy in the treatment of bloodstream infections is one of the most critical determinants of patient survival [10,11,12]. The data obtained in this study demonstrate that integrating the rapid Sepsityper^®^ kit with the EUCAST rapid antimicrobial susceptibility testing (RAST) protocol, which requires an incubation period of 4 to 20 h rather than yielding instant results, can potentially reduce laboratory turnaround time by 24–48 h compared with the conventional workflow [13,14,15]. The rapid Sepsityper^®^ kit showed considerable potential in clinical microbiology practice, providing species-level identification within 1 h in 71.8% of positive blood cultures. Identification success rates between 75% and 90% have been reported for the Sepsityper^®^ kit in the literature [16,17], and rapid MALDI-TOF MS-based identification combined with stewardship intervention has been associated with shorter time to effective therapy [18]. In our study, the identification rate was higher for Gram-negative bacilli (76.1%) than for Gram-positive cocci (64.0%). The thick peptidoglycan cell wall of Gram-positive bacteria, which impedes protein extraction, together with the lower bacterial load, is considered the main reason for the lower identification success in this group [19,20]. When the time-dependent performance of the EUCAST RAST protocol was examined, overall categorical agreement increased from 67.5% at 4 h to 87.0% at 16–20 h. However, this final rate remains slightly below the 90% threshold regarded as acceptable by EUCAST [21]. The major error (ME) rate of 51.3% at early time points, particularly at 4 h, is strikingly high. This is most likely a technical artifact arising from bacteria not yet having entered the exponential growth phase, insufficient microcolony formation, and consequently inhibition zones that have not yet fully developed [22]. As bacterial growth became more pronounced, the ME rate fell to 3.8%. Therefore, results reported as “resistant” at 4 h should be interpreted with caution and confirmed at later readings, rather than prompting immediate broadening of therapy. In contrast, the persistence of very major errors (VME), the clinically most hazardous error type, at 8.1% even at the final reading is an important warning. VME cases clustered predominantly in *K. pneumoniae* and *E. coli* isolates, particularly for carbapenems such as imipenem and meropenem [13]. In carbapenemase-producing Gram-negative bacilli (KPC, NDM, OXA-48, etc.), weak expression of the resistance phenotype at low inocula, or during direct disk diffusion in the early hours, can produce falsely wide inhibition zones and lead to misclassification as “susceptible” [23,24]. Because reporting a carbapenem-resistant strain as susceptible by RAST may result in inadequate treatment and worsen septic shock, carbapenem-susceptible RAST results should be confirmed using standard methods or rapid molecular carbapenemase tests [25]. The low categorical agreement of *S. aureus* isolates at 4 h (23.1%), which reached 93.3% at 16–20 h, clearly shows that final decisions should not be based on 4 and 6 h readings, given the slow growth kinetics of Gram-positive cocci [26]. In conclusion, the rapid Sepsityper^®^ kit can substantially accelerate the transition to targeted antimicrobial therapy by enabling species-level identification directly from positive blood cultures [27]. The method provides reliable species-level identification in approximately 80% of positive blood cultures, with results available within approximately 30 min, substantially shortening the time to microbial identification compared with conventional workflows [28]. Categorical agreement in the EUCAST RAST method is directly proportional to incubation time, rising from 67.5% to 87.0% [8]. Major errors at early time points may stem from transient technical limitations The VME rate of 8.1% at the final reading carries the risk of overlooking carbapenem resistance, particularly in *K. pneumoniae* strains [22]. Although the final VME (8.1%) and ME (3.8%) rates improved over time, the VME rate remained above generally accepted performance criteria for antimicrobial susceptibility testing. This is particularly important because VMEs represent false-susceptible results and may lead to inappropriate therapy. Therefore, RAST results should be interpreted cautiously, especially when clinically important resistance mechanisms are suspected, and discrepant or critical results should be confirmed using conventional susceptibility testing or another validated reference method [29].

### Study Limitations

Our study has several limitations that should be acknowledged. First, although the implementation of rapid identification and susceptibility testing protocols significantly reduces laboratory turn-around time (TAT), this study was primarily designed as a diagnostic workflow evaluation and did not directly assess clinical outcomes such as intensive care unit (ICU) length of stay, overall survival, or the direct impact on optimizing initial empirical antimicrobial therapy in accordance with Surviving Sepsis Campaign (SSC) guidelines [30]. Second, a formal cost-effectiveness or economic impact analysis of incorporating Sepsityper and EUCAST RAST workflows into routine laboratory operations was not performed, which represents an important area for future prospective health economic investigations. Third, the study is constrained by its single-center design and a relatively modest sample size, limiting the generalizability of our findings to institutions with different local epidemiological profiles, resistance patterns, and blood culture system infrastructures. Furthermore, the selection of the seven evaluated organism groups and specific antibiotic agents (including broad-spectrum combinations) was based on routine hospital surveillance priorities rather than an exhaustive panel, and detailed patient-level chronological data regarding prior antimicrobial exposure were beyond the scope of this workflow analysis. Fourth, automated systems (such as VITEK-2 or BD Phoenix) used in routine workflows were relied upon as the reference standard rather than employing reference broth microdilution (ISO 20776-1) [31]. or molecular resistance testing to confirm controversial results. Given that automated systems may exhibit misclassifications for carbapenems and fluoroquinolones, some of the very major errors (VMEs) or major errors (MEs) observed in the RAST evaluation might partly reflect inherent limitations or inaccuracies within the automated reference systems themselves, alongside inoculum and growth-phase biological factors. Although our final VME (8.1%) and ME (3.8%) rates show a time-dependent improvement, VME rates remain above the strict acceptable limits defined by ISO 20776-2 (typically <1.5–3%) [32], emphasizing that RAST cannot yet replace conventional gold-standard methods for critical resistance markers. Regarding species identification, MALDI-TOF MS scores were interpreted using standard manufacturer library thresholds, and unconfirmed lower-score identifications were not subjected to secondary molecular sequencing. Finally, an additional limitation concerns the statistical structure of the dataset. Although multiple antibiotic–isolate comparisons (2094 total comparisons) were generated across the different RAST reading times, these observations originated from the same 71 clinical isolates and therefore introduce a data clustering effect, meaning observations within the same isolate are not entirely independent. In addition, the number and composition of evaluable antibiotic–isolate comparisons varied between reading times due to technical read-readiness and protocol specifications. Consequently, the performance estimates should be interpreted as descriptive rather than as independent statistical comparisons between time points, and terms denoting formal statistical significance have been avoided. Moreover, the relatively small numbers of isolates for some species limit the precision of species-specific estimates; therefore, findings from small subgroups should not be generalized beyond the study population. Finally, the observed concentration of very major errors in *K. pneumoniae* strains regarding carbapenems should be interpreted in the context of our local epidemiological prevalence of carbapenemase-producing clones rather than an inherent universal failure, and potential pre-analytical inoculum density variations must be considered alongside biological growth kinetics.

## 5. Conclusions

The rapid Sepsityper^®^ kit provides successful species-level identification in positive blood culture samples in less than 1 h, making a significant contribution to the rapid narrowing of empirical therapy. Although the EUCAST RAST protocol offers categorical agreement that increases in parallel with the incubation time (87.0%), validating early reading results with conventional methods and molecular tests is of critical importance due to the risk of very major errors detected in the evaluation of carbapenem resistance, particularly in *K. pneumoniae* isolates. Our study has certain methodological limitations. First, its single-center design restricts the generalizability of the obtained resistance and error rates to different geographic regions or hospital profiles. Second, although the overall number of isolates was sufficient, the small sample size in certain subgroups, such as *P. aeruginosa* and *E. faecalis*, limited the statistical power of the RAST performance data for these species. Finally, lack of genotypic characterization of the underlying resistance genes underlying the phenotypically detected discrepancies and very major errors constitutes another limitation of the study.

## Figures and Tables

**Table 1:** Distribution of isolates included in the study by species.

Bacterial Species/Group	Number of Isolates (n)	Distribution Rate (%)
*Klebsiella pneumoniae*	22	31.0%
*Staphylococcus aureus*	15	21.1%
*Escherichia coli*	12	16.9%
*Acinetobacter* spp.	9	12.7%
*Enterococcus faecium*	6	8.5%
*Enterococcus faecalis*	4	5.6%
*Pseudomonas aeruginosa*	3	4.2%
Total	71	100%

Note: a total of 71 unique isolates from positive blood cultures were evaluated. Gram-negative bacilli predominated, with *Klebsiella pneumoniae* (*K. pneumoniae*) presenting as the most frequent species (*n* = 22, 31.0%), followed by *Staphylococcus aureus* (*S. aureus*) (*n* = 15, 21.1%) and *Escherichia coli* (*E. coli*) (*n* = 12, 16.9%). The remaining isolates were *Acinetobacter* spp. (*n* = 9), *Enterococcus faecium* (*E. faecium*) (*n* = 6), *Enterococcus faecalis* (*E. faecalis*) (*n* = 4), and *Pseudomonas aeruginosa* (*P. aeruginosa*) (*n* = 3).

**Table 2:** Species-level identification performance of the rapid Sepsityper^®^ kit.

Isolate Group/Genus	Total Isolates (n)	Successful Species Identification (n)	Failed/Low Score (n)	Identification Success Rate (%)
Gram-Negative Bacilli (*K. pneumoniae*, *E. coli*, *Acinetobacter*, *P. aeruginosa*)	46	35	11	76.1%
Gram-Positive Cocci (*S. aureus*, *E. faecium*, *E. faecalis*)	25	16	9	64.0%
Total	71	51	20	71.8%

Note: species-level identification success rates achieved directly from positive blood culture bottles using the rapid Sepsityper^®^ kit and MALDI-TOF MS.

**Table 3:** Overall categorical agreement and test numbers according to time.

Reading Time	Evaluable Tests (n)	Concordant Tests (n)	Discordant Tests (n)	Overall Categorical Agreement (%)
4 h	501	338	163	67.5%
6 h	531	407	124	76.6%
8 h	531	439	92	82.7%
16–20 h	531	462	69	87.0%

Note: overall categorical agreement increased consistently with incubation time, from 67.5% (338/501) at 4 h to 87.0% (462/531) at 16–20 h.

**Table 4:** Categorical agreement rates (%) at 4, 6, 8, and 16–20 h according to species.

Bacterial Species (n)	4 h (%)	6 h (%)	8 h (%)	16–20 h (%)
*Acinetobacter* spp. (*n* = 9)	93.7%	92.1%	92.1%	96.8%
*Staphylococcus aureus* (*n* = 15)	23.1%	48.9%	73.3%	93.3%
*Klebsiella pneumoniae* (*n* = 22)	78.3%	84.8%	85.2%	88.7%
*Escherichia coli* (*n* = 12)	55.8%	71.3%	83.7%	84.5%
*Pseudomonas aeruginosa* (*n* = 3) *	—	75.0%	79.2%	87.5%
*Enterococcus faecium* (*n* = 6)	41.7%	58.3%	70.8%	70.8%
*Enterococcus faecalis* (*n* = 4)	50.0%	50.0%	50.0%	50.0%

As detailed in Table 4, species-specific categorical agreement rates revealed distinct performance patterns across the evaluated incubation intervals. *Acinetobacter* spp. (*n* = 9) maintained exceptionally high agreement (>90%) across all time points, culminating at 96.8% at 16–20 h. Conversely, *S. aureus* (*n* = 15) exhibited a markedly low agreement of 23.1% at 4 h due to initial growth constraints, which increased substantially over time to reach 93.3% at the final reading. *K. pneumoniae* (*n* = 22) and *E. coli* (*n* = 12) showed steady improvements, achieving final agreement rates of 88.7% and 84.5%, respectively. While *P. aeruginosa* (*n* = 3) improved from 75.0% at 6 h to 87.5% at the final reading, with 4 h evaluations omitted per EUCAST guidelines—enterococcal species showed more variable results, with *E. faecium* reaching 70.8% and *E. faecalis* remaining constant at 50.0% at the final reading. * 4 h readings are not defined for *Pseudomonas aeruginosa* in the EUCAST guidelines.

**Table 5:** Distribution of error types and denominator composition according to reading times.

Reading Time	Ref. R (n)	Ref. S (n)	Ref. I (n)	Total Evaluable (n)	Very Major Error, n/N (%)	Major Error, n/N (%)	Other Discordant (n) ^a^	Total Discordant (n)
4 h	265	226	10	501	14/265 (5.3%)	116/226 (51.3%)	33	163
6 h	270	235	26	531	25/270 (9.3%)	55/235 (23.4%)	44	124
8 h	270	235	26	531	30/270 (11.1%)	18/235 (7.7%)	44	92
16–20 h	270	235	26	531	22/270 (8.1%)	9/235 (3.8%)	38	69

^a^ Ref., reference (automated AST) category. Other discordant, RAST results in the ATU plus discordances against an intermediate reference category; at 16–20 h, 29 ATU results (against R or S references) and 9 discordant results against an intermediate reference were achieved. ATU results against R or S references are already included in the Ref. R and Ref. S denominators and are not additive to them. Total discordant = VME + ME + other discordant. Note: major errors fell from 51.3% at 4 h to 3.8% at 16–20 h, whereas very major errors peaked at 11.1% at 8 h and remained at 8.1% in the final reading. Other discordant results were relatively stable (33–44 per time point). At 16–20 h, 69 of 531 evaluable tests were discordant (22 VME, 9 ME, 38 other), and 462 were concordant.

**Table 6:** Distribution of very major errors detected at the 16–20 h reading by species and antibiotics.

Bacterial Species	Antibiotic Agent	Number of Very Major Errors (n)	Clinical Significance/Comment
***K. pneumoniae***	Imipenem	3	Risk of treatment failure in carbapenemase producers
***K. pneumoniae***	Meropenem	2	Risk of treatment failure in carbapenemase producers
***K. pneumoniae***	Ciprofloxacin	3	Overlooking fluoroquinolone resistance
***K. pneumoniae***	Levofloxacin	2	Overlooking fluoroquinolone resistance
***K. pneumoniae***	Amoxicillin–clavulanic acid	2	Inhibitor-resistant beta-lactamases (e.g., OXA-1)
***K. pneumoniae***	Piperacillin–tazobactam	1	Enzyme overexpression/inhibitor resistance
***K. pneumoniae***	Gentamicin	1	Aminoglycoside resistance overestimation
***K. pneumoniae***	Ceftazidime–avibactam	1	Illusion with novel inhibitor combinations
***K. pneumoniae***	Trimethoprim–sulfamethoxazole	1	Overlooking folate pathway resistance
***E. coli***	Imipenem	1	Missing carbapenem non-susceptibility
***E. coli***	Meropenem	1	Missing carbapenem non-susceptibility
***E. coli***	Piperacillin–tazobactam	1	Missing beta-lactam/inhibitor resistance
***Acinetobacter*** **spp.**	Amikacin	1	Aminoglycoside resistance oversight
***S. aureus***	Clindamycin	1	Inducible clindamycin resistance (D-test required)
***E. faecalis***	High-Dose Gentamicin	1	Loss of high-level aminoglycoside synergy
**Total**	-	22	Critical Errors in Clinical Treatment Management

Note: distribution of very major errors (VMEs) observed at the 16–20 h reading by species and specific antimicrobial agents.

## Data Availability

The original contributions presented in the study are included in the article; further inquiries can be directed to the corresponding author.

## Abbreviations

AST	Antimicrobial Susceptibility Testing
ATU	Area of Technical Uncertainty
CA	Categorical Agreement
EUCAST	European Committee on Antimicrobial Susceptibility Testing
HCCA	Alpha-cyano-4-hydroxycinnamic acid
ICU	Intensive Care Unit
MALDI-TOF MS	Matrix-Assisted Laser Desorption/Ionization Time-of-Flight Mass Spectrometry
ME	Major Error
MHA	Mueller–Hinton Agar
RAST	Rapid Antimicrobial Susceptibility Testing
TAT	Turnaround Time
VME	Very Major Error
